# Risks to emergency medical responders at terrorist incidents: a narrative review of the medical literature

**DOI:** 10.1186/s13054-014-0521-1

**Published:** 2014-09-24

**Authors:** Julian Thompson, Marius Rehn, Hans Morten Lossius, David Lockey

**Affiliations:** London’s Air Ambulance, The Helipad, Royal London Hospital, Whitechapel Road, Whitechapel, London, E1 1BB UK; Department of Research and Development, Norwegian Air Ambulance Foundation, Holterveien 24, 1448 Drøbak, Norway; Field of Pre-hospital Critical Care, Network of Medical Sciences, University of Stavanger, Kjel Aarholmsgate 41, 4036 Stavanger, Norway; School of Clinical Sciences, University of Bristol, 69 St Michael’s Hill, Bristol, BS2 8DZ UK

## Abstract

As the threat of international terrorism rises, there is an increasing requirement to provide evidence-based information and training for the emergency personnel who will respond to terrorist incidents. Current major incident training advises that emergency responders prioritize their own personal safety above that of the ‘scene and survivors’. However, there is limited information available on the nature of these threats and how they may be accurately evaluated. This study reviews the published medical literature to identify the hazards experienced by emergency responders who have attended previous terrorist incidents. A PubMed literature search identified 10,894 articles on the subject of ‘terrorism’, and there was a dramatic increase in publications after the 9/11 attacks in 2001. There is heterogeneity in the focus and quality of this literature, and 307 articles addressing the subject of scene safety were assessed for information regarding the threats encountered at terrorist incidents. These articles demonstrate that emergency responders have been exposed to both direct terrorist threats and environmental scene hazards, including airborne particles, structural collapse, fire, and psychological stress. The emphasis of training and preparedness for terrorist incidents has been primarily on the direct threats, but the published literature suggests that the dominant causes of mortality and morbidity in responders after such incidents are the indirect environmental hazards. If the medical response to terrorist incidents is to be based on evidence rather than anecdote, analysis of the current literature should be incorporated into major incident training, and consistent collection of key data from future incidents is required.

## Introduction

Terrorist attacks have the aim of causing disruption and widespread fear [1]. In recent years, such attacks have increasingly been designed to cause maximal casualties, and sometimes emergency responders may be targeted [2-4]. Early in the emergency response to a terrorist attack, the exact intent, scale, and hazards of an incident may be unclear [5,6]. Emergency medical service (EMS) responders have occupational fatality rates that are comparable to those of other emergency services [7] and, despite major incident training that advocates prioritization of personal safety above that of the ‘scene and survivors’ [8], continue to die at terrorist incidents [9,10].

**Figure 1:**
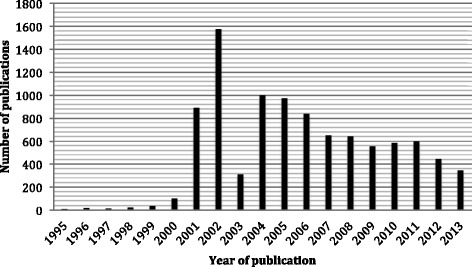
**PubMed publications on the subject of terrorism from 1995 through 2013.**

Without an informed appreciation of the potential threats, an emergency responder is unable to evaluate the risks to personal safety on arrival at a terrorist incident [11]. A rapidly expanding medical literature addresses the increasing burden of international terrorist incidents [12-14] (Figure 1), but articles that do directly address scene hazards are mostly anecdotal or based on expert opinion [15,16], extrapolate a generic approach to safety from single incidents [17], or focus on single threat types that may not be apparent to the responder on arrival at an incident [18]. Few attempts have been made to integrate information from the numerous incidents that occur each year [19,20].

This narrative review was conducted to identify hazards to EMS providers involved in the immediate management of terrorist incidents described in the medical literature. The review has the objective of using the risks or harm identified to inform emergency medical responders of likely sources of threat and allow them to mitigate risks to safety at future terrorist incidents.

## Methods

### Search strategy

**Figure 2:**
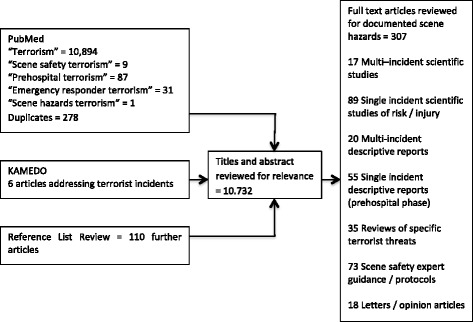
**Flow diagram of literature search and retrieved results.** KAMEDO, Swedish disaster medicine study organization.

The controlled vocabulary of Medical Subject Headings (MeSH) does not include specific terms relevant for this topic. Accordingly, we searched the electronically indexed database PubMed by using the non-indexed ‘All Field’ terms of ‘scene AND safety AND terrorism’, ‘prehospital AND terrorism’, and ‘emergency AND responder AND terrorism’. Furthermore, we applied an ‘All Field’ search of PubMed by using the term ‘terrorism’ to avoid exclusion of relevant articles (Figure 2). The search was last undertaken on 4 February 2014. Literature was limited to English language publications. The reference lists of publications found on the search criteria were scanned to identify additional relevant literature. Additionally, the reports of KAMEDO (Swedish disaster medicine study organization) were included where reporting specifically on terrorist incidents. The search was conducted independently by two authors (JT and MR).

### Selection criteria

Scoping searches revealed that few articles primarily addressed the subject of scene safety at terrorist incidents, but relevant information was included in many other articles that described the response to terrorist incidents. Therefore, no single article type was specified as an inclusion criterion, and important information was identified in study designs ranging from multi-incident studies of scene hazards through detailed single incident reports to opinion articles (Figure 2).

### Data extraction and quality appraisal

All relevant full-text articles were retrieved and assessed for the identification of risks or injury to emergency responders attending terrorist incidents. Scene hazards identified were categorized into direct terrorist threats aimed at EMS providers and, secondly, the environmental hazards of working at an unstable scene. Data on the nature of the threat and risk to emergency responders were extracted. We evaluated manuscript category of the included articles, and no further assessment of methodological quality was made.

## Results

The results of the literature search are illustrated in Figure 2.

## Threats directed at emergency responders

### Secondary explosive devices

Secondary explosive devices are bombs that are placed to cause casualties among emergency personnel responding to incidents. A delay in detonation following a primary explosion or other event is designed to draw in and then target emergency personnel in large numbers and maximize injury and damage to the emergency infrastructure [21,22]. Terrorist use of explosive devices has increased significantly in recent years, with an estimated fourfold increase worldwide from 1999 to 2006 and with an eightfold increase in related injuries [14]. Even in a country with a low risk of terrorist incidents, such as the US, 36,110 bombing incidents occurred between January 1983 and December 2002, resulting in 5,931 injured and 699 deaths, and the majority of injuries and deaths were caused by primary explosive devices with homicidal intent targeting residential or commercial locations [23]. This analysis of Bureau of Tobacco, Alcohol, Firearms, and Explosives data did not identify whether these victims were emergency medical responders but did note an increasing terrorist tendency to attempt to psychologically intimidate a country’s civilian population rather than target government resources.

The threat of secondary explosive devices creates great concern among emergency responders [5,22], and emergency responders have been exposed to numerous incidents of improvised explosive device in non-terrorist situations [24]. However, despite the increasing worldwide prevalence of primary explosive devices [14], remarkably few accounts in the medical literature pertain to secondary devices designed to target civilian emergency responders at terrorist incidents [23,25]. Secondary devices may be more frequent in asymmetrical military conflicts in which a military emergency response may be perceived by the terrorist as a legitimate military target [26-28]; however, incidents of this type are not routinely reported in the medical literature.

Perhaps of comfort to the civilian emergency responder is that of the 36,110 bombing incidents identified in the US between 1983 and 2002 [23], only four or five involved secondary devices; of this limited number, two were undertaken by the same individual: in 1997, Atlanta Olympic Park bomber Eric Rudolph planted several devices, including a device that detonated 1 hour after a primary device, injuring four public safety personnel with shrapnel and blast injuries [25].

Further reassurance may be derived from analysis of incidents where secondary devices to target rescuers have been reported. The discovery of four unexploded devices during the aftermath of the Madrid train bombings of 2004 required repeated evacuation of the incident site and rapid evacuation of casualties and has been cited as an attempt to target the emergency response [6]. However, the KAMEDO analysis of the Madrid bombings reports that the four unexploded devices had mobile phone timer triggers identical to the 10 devices that did explode but that the trigger mechanism appears to have been set exactly 12 hours out of synchronization [29]. It may be that the four unexploded devices had been accidentally allocated a different detonation time rather than an intentionally delayed detonation to target responders. Similarly, the KAMEDO report of the Bali bombing of 2002 challenges popular belief of a planned delay between the suicide vest explosion in a packed tourist bar and a minivan explosion on the street outside. It was speculated that this delay was intended to maximize casualties among fleeing revelers and arriving responders [30]. However, KAMEDO reports that the interval between the explosions may have been seconds rather than minutes as originally suspected [31]. These facts do not diminish the catastrophic loss of life in these events but may help counter fears that terrorists are routinely targeting civilian emergency responders.

### Small arms fire

Rifles and handguns have been widely used as tools of terrorism across the world and may be used alone or in conjunction with explosive devices [2,32]. In the US, around 30,000 people are killed each year by guns, and such numbers dwarf fatalities due to biological and other feared but rare modes of terrorism. Deaths due to small arms fire in the US are largely secondary to criminal rather than terrorist activity and present a significant threat to civilian emergency medical responders undertaking their duties in this country [33]. However, in regard to terrorist incidents specifically, there are few accounts of injuries to civilian responders from small arms fire in the medical literature. The majority of reports and analysis are derived from the Israel-Palestine conflict [27,34].

The perceived threat of small arms incidents dramatically increased following the 2008 attacks in Mumbai, where terrorists used multiple strategies of attack (including automatic weapons), roamed across the city, and remained active for several days. Although the 172 killed and 304 injured are not reported to have been medical personnel, civilians and security forces were killed. This generated worldwide media attention and international fear of repeated ‘Mumbai-style’ attacks [35].

The horrific massacre of at least 348 people, including 171 children, at a school in Beslan, North Ossetia (Russia), in 2005 included the murder of two paramedics by small arms fire. During negotiations with the terrorists, an agreement was made that Ministry of Emergency Situations medical personnel in two ambulances could safely remove the bodies of 20 murdered hostages from the school grounds. As the paramedics approached the school, the hostage-takers opened fire, killing two rescuers [36].

Although no emergency responders were injured in the 2011 shootings on Utoya island in Norway, the emergency responders were under direct threat during the events. During the establishment of the major incident infrastructure, the initial casualty clearing station came under direct rifle fire from the terrorist. In the same incident, when the victims were being medically assessed in what remained an unsafe environment, medical personnel were not equipped in the same way as other emergency response teams at the scene with appropriate personal protective equipment (PPE) [32].

### Chemical hazards

Despite the Chemical Weapons Convention, which came into force in 1997 and which forbids the possession, development, and use of chemical weapons, chemical terrorism remains a threat. Countries that possessed chemical weapons undertook to destroy them, but several states, including Iran, Iraq, Libya, and Israel and its neighboring countries, did not accede to the convention [37]. Even without state support, non-governmental groups or even individuals can successfully manufacture chemical weapons and may remain undetected by government intelligence agencies. Some chemical agents are used in a controlled environment in industry or agriculture and do not require illicit manufacture. Consequently concerns have been expressed regarding the accessibility of agents such as organophosphates and the vulnerability to attack or theft of chemical establishments [38,39].

In conflict and peacetime, there have been numerous chemical incidents with large numbers of casualties. There have been multiple attempts by terrorists to harness the lethal effect of such agents, but despite generating widespread fear, these attempts have met with limited success to date [37]. Secondary contamination of medical personnel treating contaminated victims is seen to occur in accidental incidents, and one study identified 17 medical personnel injured in this way over a 3-year period in the US [40].

The 1994 sarin nerve agent attacks by the Aum Shinrikyo cult in Matsumoto and Tokyo, Japan, claimed the lives of 19 people and injured over 6,000 [41-55]. Health-care workers suffered secondary contamination in both incidents; 18 were affected in Matsumoto [46] and 245 in Tokyo [44,46,48,51]. Identification of the chemical agent was delayed, and contaminated patients were treated on the scene and in the hospital by staff without appropriate PPE. Consequently, secondary contamination of medical staff occurred; in one report, 13 of 15 doctors involved in resuscitating a patient became symptomatic, 6 of whom required atropine [47]. Follow-up of victims after this sarin attack demonstrated chronic decline of psychomotor and memory function at 7 years [43] and high levels of post-traumatic stress disorder (PTSD) at 10 years [56]. The lessons learned from these incidents have informed chemical terrorism preparedness across the world [57].

### Biological hazards

Biological warfare (but not research into defense or protection against biological agents) was outlawed by the Biological Weapons Convention in 1972. Biological weapons are biological toxins or infectious agents such as bacteria, viruses, fungi, or parasites intended to kill or incapacitate and have been widely used throughout history [37,58]. With the exception of some rapidly acting toxins, biological agents usually present only hours or days after exposure with non-specific ‘flu-like’ symptoms before organ-specific diseases become apparent [58]. The risk presented by biological agents can be classified by their individual pathogenicity, infectivity, latency, lethality, transmissibility, and virulence. The US Centers for Disease Control and Prevention categorize agents depending on the threat that they may pose to national security because of their dissemination, person-to-person transmission, high mortality rates, potential for social disruption, and need for public health preparedness. The Category A (highest priority) organisms are rarely seen in the US and include anthrax, botulism, plague, smallpox, tularemia, and viral hemorrhagic fever [59]. Category B agents are more commonly encountered and include food-and-water safety threats such as Salmonella species, *Escherichia coli* 0157:H7, and *Vibrio cholerae*. Ricin is the Category B agent most frequently encountered in the US, can be easily prepared from castor beans, and has been used in ‘white powder’ letters. Although such acts have been largely criminal in nature rather than true bioterrorism and are frequently hoaxes, such incidents pose a potential threat to emergency medical responders [60]. Successful terrorist use of biological weapons is extremely rare, and one source suggests that only two confirmed terrorist biological attacks have harmed humans [61].

Between October and December 2001, widespread fear was caused across the US by a series of letters containing anthrax spores that were sent to government buildings. Five people died from anthrax, 13 contracted disease, and many thousands were exposed and took preventative antibiotics. Health-care personnel were not specifically targeted, although other emergency services required to deal with suspicious packages were exposed to risk [62].

The difficulty in identifying biological attacks is apparent from the Salmonella typhi outbreak in The Dalles, Oregon, in 1984 when 751 citizens were affected [63]. Only 1 year later did it emerge that the Rajneeshee cult had intentionally contaminated water and salad bars in an attempt to influence a local election result. Similarly, when the Aum Shinrikyo cult was investigated in the wake of the 1995 Tokyo sarin subway attacks, it was discovered that they had built three laboratories to culture *Bacillus anthracis*, botulinum toxin, and *Coxiella burnetti* and carried out nine undetected biological weapon attacks between 1990 and 1993 [58,64].

Secondary biological threat has been identified as a consequence of exposure to contaminated biological material in explosive incidents. Following the London bombings of 7 July 2005, bone fragments from other victims were found embedded as biological foreign bodies within the soft tissues of five patients at one receiving hospital [65]. Similar events have occurred in suicide bombings in Israel and in conflict zones against US military personnel, and protocols have been established for post-exposure interventions to prevent infection with hepatitis B and C, HIV, or tetanus [66-68].

### Radiation

Nuclear detonation by terrorists is perceived to be unlikely given the state-sponsored level of technology required to develop or deploy a device [37]. A ‘dirty bomb’ or radiological dispersal device (RDD) is a more likely scenario [69]. Radiation sources are routinely used in science, industry, and medicine and could be used by terrorists to create an RDD. Concerns have been compounded by the low level of security surrounding these sources, and there is documented evidence of multiple missing sources [70-72].

A very small amount of radioactive energy can cause serious biological damage. External radiation is primarily gamma radiation that has no mass, travels long distances in air, and penetrates shielding. Alpha and beta particles represent the dominant risk if a radioactive substance has entered the body. Alpha particles consist of two neutrons and two protons, and although they can travel up to 3 cm in air, they cannot penetrate skin but are extremely dangerous if ingested or inhaled. Beta particles are electrons that can penetrate approximately 5 mm in skin and 3 cm in air [73].

In recent decades, there have been multiple radiation accidents, including incidents at nuclear power stations and the accidental misuse of scientific radiation sources [37,74]. The single non-accidental incident using radiation identified in the medical literature is an assassination that occurred in London in 2006, when Alexander Litvinyenko was poisoned with polonium-210. The single victim is alleged to have unknowingly ingested an alpha source and died 22 days later suffering from multiorgan failure as a result of radiation. Despite the limited size of this polonium-210 source, the potential scale of the radiation contamination was illustrated by the 664 individuals from 52 countries who were considered at risk of exposure following this single incident [75].

## Secondary threats for emergency responders

### Environmental hazards

Although chemical, biological, radiological, nuclear, and explosive (CBRNE) hazards are correctly emphasized when terrorist incidents are discussed, the more conventional scene hazards of working in an unstable environment may be associated with greater risk [76]. The list of potential sources of danger at an incident is lengthy. However, one generic approach to risk identification is that of identifying and mitigating the threat of unstable energy sources, most commonly in the form of kinetic, potential, electrical, or thermal energy [11]. This theoretical exercise can identify the key hazards of many high-risk environments but may be of limited practical assistance when confronted by a mass casualty terrorist incident.

If the medical literature is assessed to determine the main environmental threats at terrorist incidents, the majority of the relevant publications assess the aftermath of the 9/11 attacks, where extraordinary resources were required both in the initial phase of the response and in the prolonged process of rescue and recovery. Unprecedented numbers of rescuers worked at the site, and their experience has created an unparalleled resource documenting the medical sequelae of responding to such an incident. Between 14 and 21 November 2001, a medical center was established at the scene of the World Trade Center (WTC) attacks and treated 9,349 rescue and recovery personnel working on the site. The most common presentations were traumatic injuries (29%), respiratory problems (22%), and eye complaints (12%). The medical literature published following the 9/11 attacks and other international terrorist incidents has illustrated several principal immediate hazards and long-term consequences.

### Airborne particles

Explosions, fire, and building collapses as a consequence of terrorist activity create dust clouds containing particulate matter that may have an immediate and a delayed health impact for emergency responders. This effect has been most extensively studied following the 9/11 attacks [77]. Although no air-sampling monitors were operating close to the WTC on the day of this attack, analysis of fallen dust samples was performed 5 and 6 days afterward. This demonstrated contamination (1% to 4% by weight) with particles small enough to be respirable in rescue workers without protective masks [78]. Early symptoms reported included eye, skin, respiratory, and nose and throat complaints [79].

Extensive longitudinal study of WTC workers has been undertaken and has demonstrated that this cohort has an increased incidence of asthma, sinusitis, chemosensory loss, sarcoidosis, and gastroesophageal reflux disease [80-83]. Additionally, suspected carcinogens in the dust cloud have been proposed as the cause of excess risk for prostate cancer, thyroid cancer, and myeloma in WTC rescue workers when compared with that for New York State residents [84]. Appropriate respiratory protection reduces the risk of the respiratory and systemic manifestations of airborne pollutants [85]. In emergency responders to the 9/11 attacks, longer delays in the initial use of masks or respirators were associated with an increased risk of asthma, as was earlier arrival and longer duration of exposure [86].

### Structural instability

Building collapse has been proposed as the most important risk factor for fatality in building bombings [87], and residents and responders are at risk following an incident. When the twin towers of the WTC collapsed, over 400 emergency responders lost their lives [88]. Proposals for reducing fatalities in a building bombing include evacuation planning exercises for vulnerable buildings and regular evacuation training of personnel [87,89]. Appropriate PPE, including eye protection, can reduce the risk of injury due to falling objects and flying glass [90,91].

### Fire

Fires at terrorist incidents may continue to burn for many weeks and pose an ongoing threat of both direct thermal injury and inhaled toxic agents, such as carbon monoxide and hydrogen cyanide [9,92-94]. Close communication with fire services and the avoidance of exposure unless wearing appropriate PPE are central to minimizing risk [11,92].

### Mental health

Many studies have identified psychopathology among emergency responders to terrorist incidents [95-103]. Although the prevalence of PTSD varies greatly between studies and incidents, one of the largest cohorts studied consists of the 28,962 rescue workers who worked at the WTC site. The overall prevalence of PTSD among rescue/recovery workers was 12.4%, but the risk was increased in those who arrived earlier, who worked for longer, and who were asked to undertake roles that were not within their usual professional remit [104]. A longitudinal study of a similar cohort of WTC rescue and recovery workers demonstrated a cumulative incidence of depression of 27.5%, PTSD in 31.9%, and panic disorder in 21.2% [82].

Protective factors that may reduce the risk of developing psychiatric illness following exposure to a terrorist incident include good pre-event mental health, disaster preparedness training, and shift rotations that limit the duration of an individual’s exposure to the scene [102,104]. Moreover, exhaustion and sleep deprivation have been demonstrated to impair threat detection and may compound the risk of exposure to other scene hazards [105]. Psychiatric services have recently been proposed to have an important role in disaster response planning [106], and critical incident stress debriefing has been reported to mitigate the impact of an event [107]. However, even if staff members are aware that such services exist, they are often reluctant to seek help [102,103]. Once psychiatric illness has developed, cognitive behavioral and virtual reality therapies have both been demonstrated to reduce symptom scores in PTSD following terrorist incidents [108,109].

## Discussion

To the best of our knowledge, this is the first systematic review of the medical literature that aims to identify the hazards that emergency responders have been exposed to at international terrorist incidents. The threat of CBRNE hazards exists at terrorist and non-terrorist incidents, and the risks must be actively mitigated. However, direct injury to emergency responders from such hazards is extremely rare and is dwarfed by the more conventional scene hazards that professionals encounter when responding to both terrorist and non-terrorist incidents [7,11,33,110].

This strategy of systematically seeking evidence-based information from the published medical literature is a conventional medical and scientific methodology [111]. Additionally, this approach creates clear boundaries in source materials when addressing such a security-sensitive issue as terrorism. However, on this subject, the approach and the available medical literature do have limitations. Despite the number of publications, the medical literature does not present a comprehensive account of international terrorist incidents or their medical sequelae. Medical publications do not reflect the daily terrorist attacks across the globe but rather cluster around high-profile events that occur in countries with a culture of medical publication [12,13]. Organizations that are dedicated to producing high-quality analysis and recommendations from international incidents have the capacity to report only a handful of the thousands of incidents that occur each year [37]. Even accurately establishing the denominator of total terrorist incidents remains challenging in the open-source literature, with varying global estimates and methodological difficulties in verifying data even in individual developed countries [112]. One source of worldwide data on terrorist incidents identified 19,828 incidents with 86,568 injured casualties and 25,408 fatalities between 1968 and 2004 [113], and only a small fraction of these incidents generated medical publications. Medical publications that do arise from terrorist incidents are of variable format and quality. Little standardization exists in the published incident reports, and few explicitly address issues such as scene safety, which may be of critical importance to future planning and safety.

Critical information regarding scene hazards is frequently encountered in the text of reports of the medical response to terrorist incidents but is usually incidental to the principal subject of the article. Closer examination of the included studies indicated inconsistent indexing of articles on risks to EMS responders at terrorist incidents on PubMed. Increasingly consistent reporting of studies related to EMS personnel safety may reduce these limitations.

The limited publication focus on the early medical response is reflected in the fact that 40% of disaster-related publications address the subject of mental health compared with only 4% on the subject of emergency response [13]. This may indicate the difficulties in data collection from chaotic scenes as opposed to the relative ease of psychological assessment in the long recovery phase.

Despite these limitations, the medical literature represents a large and extremely valuable source of information from hundreds of incidents that together should inform future practice. Other studies have similarly used a systematic approach to local and international incidents and derived high-quality evidence and identified recurrent systematic problems that may improve the quality and safety of future response [19,20,23,114]. The results of this study may be used to further inform the provision of training and PPE for emergency services personnel who may be expected to respond in the early stages of terrorist incidents.

If the medical response to terrorist incidents is to be based on evidence rather than anecdote, then further analysis of the literature and future incidents should be undertaken. However, to achieve this, there is a critical need to improve international data collection. Several groups have published standardized medical reporting templates for major incidents but, despite their quality as research tools, they are not being routinely used [115,116]. If the focus is to be on learning from the growing international burden of terrorist incidents across the world, then a medical reporting mechanism that does not have the high barrier that conventional medical publication presents needs to be established. A template for reporting pre-hospital major incident medical management has recently been developed following a consensus process of European major incident experts [117]. This template has been incorporated into an open-access website [118], allowing reporting of critical information that may help save the lives of victims and responders in future terrorist incidents.

## Conclusions

Current major incident training emphasizes the importance of personal safety but is unable to provide an evidence-based analysis of the scene hazards encountered at terrorist incidents. There is a need to refine safety guidance for emergency medical responders in light of the experience from the thousands of international incidents that occur each year. The medical literature represents an incomplete and inconsistent record of the global burden of terrorist incidents but reports a diverse range of threats at previous incidents. Interestingly, while the direct terrorist threats of CBRNE constitute the principal focus of major incident training and the predominant fears of responders, the conventional scene hazards of building collapse, airborne particles, and mental health sequelae continue to cause greater harm to emergency responders. If the medical response to terrorist incidents is to be based on evidence rather than anecdote, analysis of the current literature should be incorporated into major incident training. Of critical importance is the need to improve data collection from major incidents so that the emergency response can develop an evidence-based approach to saving the lives of victims and responders in the future.

## Abbreviations

CBRNE: Chemical, biological, radiological, nuclear and explosive
EMS: Emergency medical service
KAMEDO: Swedish disaster medicine study organization
PPE: Personal protective equipment
PTSD: Post-traumatic stress disorder
RDD: Radiological dispersal device
WTC: World Trade Center
